# Supplementary material for: Major Active Metabolite Characteristics of Dendrobium officinale Rice Wine Fermented by Saccharomyces cerevisiae and Wickerhamomyces anomalus Cofermentation
Source: Foods. 2023 Jun 14;12(12):2370. doi: 10.3390/foods12122370 (PMC10297114; doi:10.3390/foods12122370)
Supplement: Supplementary file 1 [file foods-12-02370-s001.zip › foods-2407619-supplementary.pdf]

## Supplementary

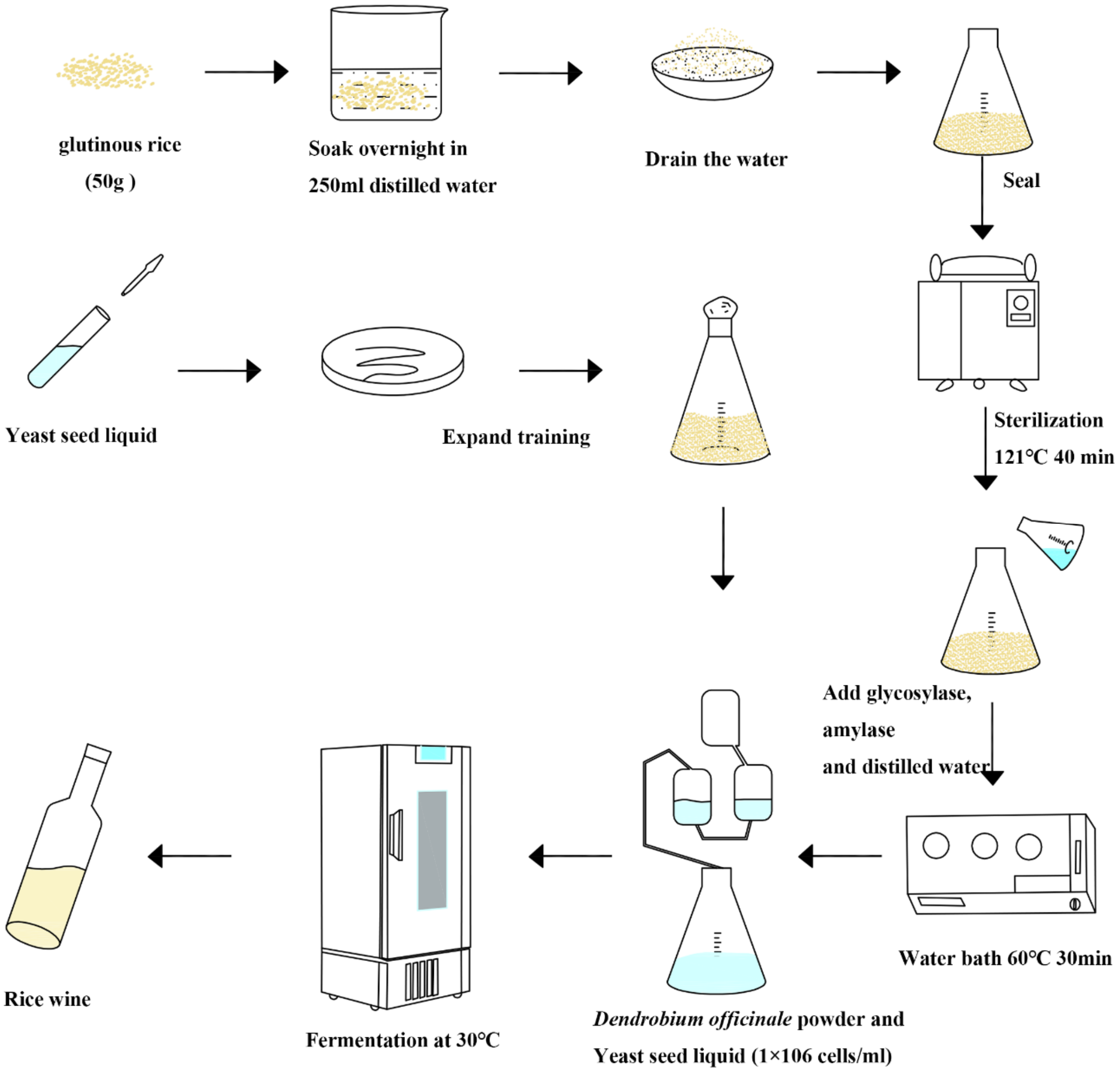


**Figure S1.** Flowchart of the fermentation process.

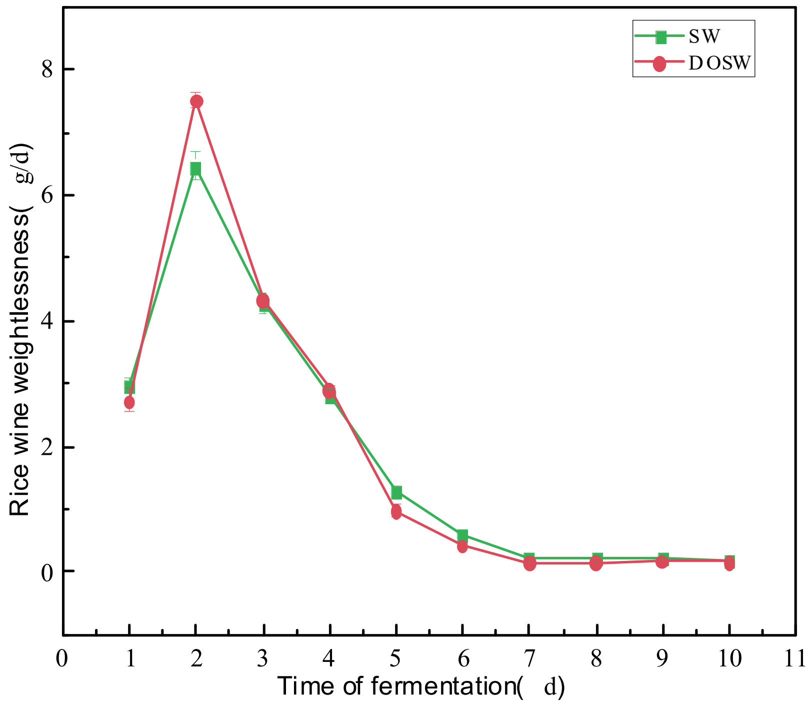


**Figure S2.** Changes of $CO_2$ weight loss in rice wine.

**Table S1.** Sensory evaluation of *Dendrobium officinale* rice wine.

| Projects | 1-9 points | 10-19 points | 20-25 points |
|---|---|---|---|
| Appearance | With the product should be colored, packaging containers with a large amount of sediment at the bottom, with foreign impurities | The product should be colored, the packaging container bottom with obvious precipitates, and no foreign impurities | The product should be colored, the packaging container bottom with a trace of sediment, and no foreign impurities |
| Fragrance | Slight aroma characteristic of rice wine | Mellow aroma characteristic of rice wine | Rich rice wine with the characteristic mellow aroma, no foreign aroma |
| Taste | Poor taste, has a strange taste | Good taste, no unpleasant taste | Mellow, sweet, crisp, harmonious, no unpleasant taste |
| Style | Almost unfermented rice wine style | Fermented rice wine style available | Clearly fermented rice wine style |

**Table S2**. Relative percentages of major active metabolites in rice wine

| Serial numb | Compounds | Classification | Ions Mode | Molecular weight | SW | DOSW | DO |
|---|---|---|---|---|---|---|---|
| 1 | 3,3',4'5-Tetrahydroxystilbene | Phenylpropanoids | N | 243.06 | 0.205±0.006[a] | 0.107±0.008[b] | 0.021±0.004[c] |
| 2 | Aspalathin | Phenylpropanoids | N | 451.12 | 0±0[b] | 0.279±0.005[a] | 0.296±0.015[a] |
| 3 | 2-Hydroxy-3-(4-methoxyphenyl)propanoic acid | Phenylpropanoids | P | 197.08 | 0.112±0.023[c] | 0.172±0.008[b] | 1.117±0.032[a] |
| 4 | 3-(3,4,5-Trimethoxyphenyl)propanoic acid | Phenylpropanoids | P | 258.13 | 1.27±0.046[a] | 0.795±0.016[b] | 0.026±0.004[c] |
| 5 | Ampelopsin D | Phenylpropanoids | P | 455.15 | 0±0[c] | 0.025±0[a] | 0.01±0.003[b] |
| 6 | Curcumin dimer 1 | Phenylpropanoids | P | 735.25 | 0±0[c] | 0.031±0.001[a] | 0.015±0.002[b] |
| 7 | Methyldopa | Phenylpropanoids | P | 212.09 | 0.165±0.012[c] | 1.168±0.023[b] | 3.751±0.516[a] |
| 8 | Secoisolariciresinol | Phenylpropanoids | P | 363.18 | 0.057±0.004[a] | 0.052±0.039[a] | 0±0[b] |
| 9 | Marmesin | Phenols | N | 245.08 | 0.624±0.149[a] | 0.404±0.024[b] | 0.014±0.018[c] |
| 10 | (-)-Matairesinol | Phenols | N | 357.13 | 0.037±0.007[b] | 0.357±0.03[a] | 0.093±0.122[b] |
| 11 | Methyl vanillate | Phenols | N | 181.05 | 1.13±0.028[b] | 1.057±0.047[b] | 2.24±0.602[a] |
| 12 | o-Cresol | Phenols | N | 107.05 | 0.989±0.111[a] | 1.013±0.055[a] | 0.131±0.042[b] |
| 13 | Acetaminophen | Phenols | N | 150.06 | 1.403±0.227[b] | 1.769±0.06[a] | 2.428±0.283[a] |
| 14 | Hydroxytyrosol | Phenols | N | 153.06 | 0.385±0.032[b] | 0.905±0.093[a] | 0.063±0.014[c] |
| 15 | 3-O-p-Coumaroylquinic acid | Phenols | N | 337.09 | 2.13±0.069[a] | 1.738±0.091[b] | 0.931±0.202[c] |
| 16 | trans-Cinnamic acid | Phenols | N | 147.04 | 14.259±0.649[b] | 15.867±0.627[a] | 12.938±0.3[c] |

| | | | | | | | |
|---|---|---|---|---|---|---|---|
| 17 | Scoparone | Phenols | P | 207.07 | 0.014±0.002$^{c}$ | 0.487±0.019$^{a}$ | 0.4±0.051$^{b}$ |
| 18 | Phlorin | Phenols | P | 289.09 | 0.402±0.009$^{a}$ | 0.337±0.011$^{b}$ | 0.102±0.007$^{c}$ |
| 19 | trans-Ferulic acid | Phenols | N | 193.05 | 22.479±0.564$^{a}$ | 22.899±0.054$^{a}$ | 0.915±0.086$^{b}$ |
| 20 | Sinapic acid | Phenols | N | 223.06 | 0.489±0.036$^{b}$ | 0.86±0.064$^{a}$ | 0.262±0.017$^{c}$ |
| 21 | Syringic acid | Phenols | N | 197.04 | 1.163±0.032$^{a}$ | 1.05±0.044$^{b}$ | 0.341±0.066$^{c}$ |
| 22 | 8-Acetoxypinoresinol | Phenols | P | 417.15 | 0±0$^{c}$ | 0.242±0.006$^{b}$ | 0.731±0.02$^{a}$ |
| 23 | Vanillic acid | Phenols | N | 167.03 | 0.663±0.518$^{b}$ | 2.093±0.195$^{a}$ | 0.991±0.028$^{b}$ |
| 24 | 2-Benzylidene-1-heptanol | Phenols | P | 205.16 | 0±0$^{b}$ | 0±0$^{b}$ | 0.005±0$^{a}$ |
| 25 | Phenyl acetate | Phenols | P | 137.06 | 0.072±0.01$^{b}$ | 0.081±0.008$^{b}$ | 0.212±0.036$^{a}$ |
| 26 | 4-Hydroxy-2,6-dimethylaniline | Phenols | P | 138.09 | 16.377±0.748$^{a}$ | 13.167±0.408$^{b}$ | 1.496±0.268$^{c}$ |
| 27 | Coniferin | Phenols | P | 579.17 | 0±0$^{b}$ | 0±0$^{b}$ | 0.005±0$^{a}$ |
| 28 | Phenol | Phenols | P | 95.05 | 0.475±0.04$^{b}$ | 0.6±0.04$^{a}$ | 0±0$^{c}$ |
| 29 | 2-Hydroxycinnamic acid | Phenols | P | 165.05 | 5.727±0.474$^{b}$ | 5.676±0.241$^{b}$ | 25.686±0.831$^{a}$ |
| 30 | Safflor Yellow A | Phenols | P | 595.17 | 0±0$^{c}$ | 0.022±0.001$^{b}$ | 0.058±0.005$^{a}$ |
| 31 | Hydroxysafflor yellow A | Phenols | P | 613.17 | 0.095±0.014$^{b}$ | 2.907±0.113$^{a}$ | 0±0$^{b}$ |
| 32 | 4-O-Caffeoylshikimic acid | Phenols | P | 337.09 | 0.052±0.007$^{c}$ | 0.129±0.006$^{b}$ | 0.18±0.036$^{a}$ |
| 33 | 3-Hydroxybenzoic acid | Phenols | N | 137.02 | 0.264±0.004$^{c}$ | 0.407±0.018$^{b}$ | 0.749±0.057$^{a}$ |
| 34 | 4-Nitrophenol | Phenols | N | 138.02 | 0.196±0.00$^{8}$ | 0.205±0.0$^{1}$ | 0±$^{0}$ |
| 35 | Occidentoside | Phenols | P | 705.18 | 0.001±$^{0}$ | 0.018±0.00$^{1}$ | 0±$^{0}$ |
| 36 | Phloretin | Phenols | P | 275.09 | 0.024±0.002$^{b}$ | 0.024±0.001$^{b}$ | 0.059±0.005$^{a}$ |
| 37 | 5,7-dihydroxy-2-(4-hydroxy-3,5-dimethoxyphenyl)-4H-chromen-4-one | Flavonoids | N | 329.07 | 0.061±0.017$^{b}$ | 0.129±0.004$^{a}$ | 0.022±0.001$^{c}$ |
| 38 | Gingerol | Flavonoids | N | 293.18 | 4.13±2.356$^{a}$ | 1.888±0.071$^{a}$ | 1.453±0.702$^{a}$ |
| 39 | Naringenin | Flavonoids | N | 271.06 | 0.03±0.029$^{b}$ | 0.451±0.023$^{b}$ | 28.074±2.753$^{a}$ |
| 40 | Daidzein | Flavonoids | P | 255.06 | 0.365±0.028$^{a}$ | 0.215±0.013$^{b}$ | 0.033±0.004$^{c}$ |
| 41 | Naringin | Flavonoids | N | 579.17 | 0±0$^{a}$ | 0.035±0.046$^{a}$ | 0.035±0.006$^{a}$ |

| | | | | | | | |
|---|---|---|---|---|---|---|---|
| 42 | Puerarin | Flavonoids | N | 415.1 | 0±0$^{b}$ | 0±0$^{b}$ | 0.014±0.001$^{a}$ |
| 43 | Licochalcone B | Flavonoids | P | 287.09 | 0.055±0.003$^{b}$ | 0.049±0.005$^{b}$ | 0.146±0.004$^{a}$ |
| 44 | Epicatechin | Flavonoids | N | 289.07 | 0.055±0.026$^{a}$ | 0.042±0.001$^{a}$ | 0.033±0.001$^{a}$ |
| 45 | Glycitein | Flavonoids | P | 285.08 | 0.012±0.004$^{b}$ | 0.008±0.001$^{b}$ | 0.018±0.001$^{a}$ |
| 46 | (+)-Gallocatechin | Flavonoids | N | 305.07 | 0.556±0.723$^{a}$ | 0.127±0.006$^{a}$ | 0.028±0.003$^{a}$ |
| 47 | Dihydrocapsaicin | Flavonoids | N | 306.21 | 0.696±0.088$^{a}$ | 0.484±0.005$^{b}$ | 0.01±0.001$^{c}$ |
| 48 | Mulberrin | Flavonoids | N | 421.16 | 0±0$^{c}$ | 0.027±0.001$^{b}$ | 0.16±0.018$^{a}$ |
| 49 | Mammeigin | Flavonoids | P | 405.17 | 0.012±0.002$^{a}$ | 0.011±0.001$^{a}$ | 0.006±0$^{b}$ |
| 50 | Isovestitol | Flavonoids | P | 273.11 | 0.17±0.014$^{a}$ | 0.097±0.005$^{a}$ | 0.294±0.219$^{a}$ |
| 51 | Hesperidin | Flavonoids | P | 611.19 | 0.058±0.006$^{a}$ | 0.033±0.001$^{b}$ | 0±0$^{c}$ |
| 52 | Isosakuranin | Flavonoids | N | 447.13 | 0.718±0.065$^{a}$ | 0.551±0.016$^{b}$ | 0±0$^{c}$ |
| 53 | Silidianin | Flavonoids | P | 485.14 | 0±0$^{c}$ | 0.014±0$^{b}$ | 0.032±0$^{a}$ |
| 54 | Licorisoflavan A | Flavonoids | P | 439.25 | 0.944±0.031$^{a}$ | 0.776±0.054$^{b}$ | 0±0$^{c}$ |
| 55 | Sakuranetin | Flavonoids | N | 461.08 | 0.018±0.003$^{b}$ | 0.05±0.006$^{a}$ | 0.006±0.001$^{c}$ |
| 56 | Poncirin | Flavonoids | P | 595.2 | 0±0$^{b}$ | 0.012±0.001$^{a}$ | 0.014±0.003$^{a}$ |
| 57 | (-)-Epiafzelechin | Flavonoids | N | 273.07 | 0.114±0.03$^{a}$ | 0.027±0.001$^{b}$ | 0.028±0.002$^{b}$ |
| 58 | Rotenone | Flavonoids | N | 393.13 | 0.006±0$^{b}$ | 0.019±0.001$^{a}$ | 0±0$^{c}$ |
| 59 | Limocitrin | Flavonoids | N | 345.06 | 0.174±0.008$^{b}$ | 0.212±0.009$^{a}$ | 0.078±0.017$^{c}$ |
| 60 | Neohesperidin dihydrochalcone | Flavonoids | P | 613.21 | 0±0$^{b}$ | 0.082±0.01$^{a}$ | 0±0$^{b}$ |
| 61 | Catechin | Flavonoids | N | 289.07 | 1.75±0.227$^{a}$ | 0.989±0.098$^{b}$ | 0.009±0.011$^{c}$ |
| 62 | Spinosin A | Flavonoids | P | 815.24 | 0±0$^{a}$ | 0.03±0.038$^{a}$ | 0±0$^{a}$ |
| 63 | Miscanthoside | Flavonoids | N | 449.11 | 0.277±0.044$^{a}$ | 0.15±0.011$^{b}$ | 0±0$^{c}$ |
| 64 | Isoginkgetin | Flavonoids | P | 581.15 | 0.154±0.005$^{b}$ | 0.222±0.008$^{a}$ | 0±0$^{c}$ |
| 65 | Biorobin | Flavonoids | N | 593.15 | 0.006±0.004$^{c}$ | 0.025±0$^{b}$ | 0.04±0.003$^{a}$ |
| 66 | Glabrone | Flavonoids | P | 337.11 | 0.08±0.003$^{b}$ | 0.104±0.002a$^{b}$ | 0.127±0.028$^{a}$ |
| 67 | Eriodictyol | Flavonoids | N | 287.05 | 0.939±0.067$^{a}$ | 0.55±0.473a$^{b}$ | 0.015±0.002$^{b}$ |

| | | | | | | | |
|---|---|---|---|---|---|---|---|
| 68 | Quercetin | Flavonoids | N | 301.03 | 0.065±0.006[a] | 0.02±0.001[b] | 0.014±0.004[b] |
| 69 | Myricetin | Flavonoids | N | 317.03 | 0.187±0.02[a] | 0.048±0.006[b] | 0±0[c] |
| 70 | 2',7-Dihydroxy-4',5'-dimethoxyisoflavone | Flavonoids | P | 315.09 | 0.1±0.01[b] | 0.078±0.005[b] | 0.27±0.065[a] |
| 71 | 2,2,6,6-Tetramethyl-4-piperidinone | Alkaloids | P | 156.14 | 0.243±0.018[b] | 0.239±0.015[b] | 0.295±0.028[a] |
| 72 | Myosmine | Alkaloids | P | 147.09 | 0.229±0.012[b] | 0.374±0.037[a] | 0.036±0.003[c] |
| 73 | Indole-3-carbinol | Alkaloids | N | 146.06 | 0.071±0.018[b] | 0.104±0.013[a] | 0±0[c] |
| 74 | Nordihydrocapsaicin | Alkaloids | P | 294.21 | 0.015±0.002[b] | 0.015±0.001[b] | 0.048±0.006[a] |
| 75 | Pterolactam | Alkaloids | P | 116.07 | 2.083±0.093[a] | 2.105±0.125[a] | 0.468±0.014[b] |
| 76 | Pyrophaeophorbide a | Alkaloids | P | 535.27 | 0.004±0[b] | 0.006±0[b] | 0.192±0.016[a] |
| 77 | Creatinine | Alkaloids | P | 114.07 | 1.104±0.088[c] | 0.762±0.094[b] | 2.888±0.666[a] |
| 78 | Tetramethylpyrazine | Alkaloids | P | 137.11 | 0±0[a] | 0.068±0.004[b] | 0.322±0.04[c] |
| 79 | Paxilline | Alkaloids | N | 434.23 | 0.055±0.033[a] | 0.036±0.006[a] | 0.116±0.011[b] |
| 80 | Biflorin | Alkaloids | P | 355.1 | 0.154±0.008[c] | 1.77±0.023[b] | 4.156±0.129[a] |
| 81 | Cepharadione B | Alkaloids | P | 322.11 | 0.043±0.005[a] | 0.035±0.002[b] | 0.001±0[c] |
| 82 | Neferine | Alkaloids | P | 625.33 | 0.002±0[b] | 0.002±0[a] | 0±0[c] |
| 83 | Oxyacanthine | Alkaloids | P | 609.3 | 0.02±0.002[b] | 0.033±0.002[a] | 0±0[c] |
| 84 | Trigonelline | Alkaloids | P | 160.04 | 1.304±0.131[a] | 0.795±1.04[a] | 0.492±0.049[a] |
| 85 | Pyrroloquinoline quinone | Alkaloids | P | 331.02 | 0.424±0.033[a] | 0.477±0.104[a] | 0±0[b] |
| 86 | Norsanguinarine | Alkaloids | P | 334.11 | 0.352±0.018[a] | 0.337±0.028[a] | 0.034±0.004[b] |
| 87 | N6-Methyladenosine | Alkaloids | P | 282.12 | 0.322±0.063[b] | 0.434±0.074a[b] | 0.548±0.119[a] |
| 88 | Theophylline | Alkaloids | N | 179.06 | 0.964±0.173[a] | 1.07±0.031[a] | 1.009±0.212[a] |
| 89 | Betaine | Alkaloids | P | 118.09 | 0.25±0.019[b] | 0.245±0.006[b] | 0.47±0.022[a] |
| 90 | Barringtogenol C | Terpenoids | P | 491.37 | 0.028±0.005[a] | 0.019±0.002[b] | 0.013±0.004[b] |
| 91 | Sciadonic acid | Terpenoids | P | 307.26 | 0.716±0.11[a] | 0.358±0.012[b] | 0.032±0.002[c] |
| 92 | Santene | Terpenoids | P | 123.12 | 0.114±0.017[b] | 0.096±0.01[b] | 0.244±0.023[a] |
| 93 | Alpha-dihydroartemisinin | Terpenoids | P | 285.17 | 0.005±0.001[b] | 0.011±0.001[a] | 0±0[c] |

| | | | | | | | |
|---|---|---|---|---|---|---|---|
| 94 | Geniposidic acid | Terpenoids | P | 375.13 | 0±0[c] | 0.379±0.006[a] | 0.034±0.007[b] |
| 95 | Ganoderic acid Mc | Terpenoids | P | 631.38 | 0.752±0.037[a] | 0.502±0.034[b] | 0.004±0.001[c] |
| 96 | beta-Santalic acid | Terpenoids | P | 235.17 | 0.004±0[c] | 0.019±0.002[b] | 0.053±0.003[a] |
| 97 | Sterebin A | Terpenoids | P | 311.22 | 4.914±0.174[a] | 2.059±0.068[b] | 0.033±0.001[c] |
| 98 | Isoeugenitol | Terpenoids | N | 205.05 | 0.542±0.121[a] | 0.424±0.051[a] | 0.03±0.004[b] |
| 99 | Ginkgolide B | Terpenoids | P | 425.14 | 0±0[c] | 0.045±0.004[b] | 0.111±0.011[a] |
| 100 | Lucidenic acid D1 | Terpenoids | P | 471.23 | 0.038±0.002[b] | 0.088±0.013[a] | 0±0[c] |
| 101 | Valerenolic acid | Terpenoids | P | 251.16 | 0.043±0.002[a] | 0.035±0.001[b] | 0±0[c] |
| 102 | beta-Costic acid | Terpenoids | P | 235.17 | 0.003±0[c] | 0.009±0.002[a] | 0.007±0[b] |
| 103 | Capsidiol | Terpenoids | P | 219.17 | 0±0[c] | 0.009±0.003[b] | 0.03±0.001[a] |
| 104 | Genipin | Terpenoids | N | 225.08 | 0.127±0.004[b] | 0.167±0.004[a] | 0.159±0.014[a] |
| 105 | Jangomolide | Terpenoids | P | 469.18 | 0.006±0.001[b] | 0.007±0[b] | 0.018±0.002[a] |
| 106 | Perillyl aldehyde | Terpenoids | P | 151.11 | 0.304±0.017[a] | 0.269±0.031[a] | 0.097±0.009[b] |
| 107 | Dihydroisoalantolactone | Terpenoids | P | 235.17 | 0±0[b] | 0±0[b] | 0.021±0.002[a] |
| 108 | Pteroside D | Terpenoids | P | 411.2 | 0.041±0.003[a] | 0.028±0.002[b] | 0±0[c] |
| 109 | Cinncassiol D1 glucoside | Terpenoids | P | 515.28 | 0.029±0.003[b] | 0.04±0.007[a] | 0±0[c] |
| 110 | Alantolactone | Terpenoids | P | 233.15 | 0±0[b] | 0.003±0[a] | 0±0[b] |
| 111 | 1,4-Dimethyl-7-ethylazulene | Terpenoids | P | 185.13 | 0.352±0.008[c] | 0.536±0.059[b] | 1.258±0.071[a] |
| 112 | Ginkgolide C | Terpenoids | P | 441.14 | 0±0[b] | 0.199±0.015[a] | 0.26±0.051[a] |
| 113 | Obacunone | Terpenoids | P | 455.2 | 0.004±0[b] | 0±0[c] | 0.012±0.002[a] |
| 114 | Lacinilene C | Terpenoids | P | 247.13 | 0.101±0.017[a] | 0.059±0[b] | 0.011±0.002[c] |
| 115 | Astaxanthin | Terpenoids | P | 597.4 | 0.026±0.002[a] | 0.011±0.002[b] | 0±0[c] |
| 116 | Cynaroside A | Terpenoids | P | 445.2 | 0.015±0.002[a] | 0.008±0.001a[b] | 0±0[b] |
| 117 | alpha-Bixin | Terpenoids | P | 395.23 | 0.099±0.009a[b] | 0.105±0.012[a] | 0.01±0.001[b] |
| 118 | Valtrate | Terpenoids | P | 423.2 | 0.107±0.009[a] | 0.075±0.007[a] | 0.002±0[b] |
| 119 | Demethyloleuropein | Terpenoids | P | 527.17 | 0±0[c] | 0.234±0.013[a] | 0.073±0.006[b] |

| | | | | | | | |
|---|---|---|---|---|---|---|---|
| 120 | Cinnzeylanol | Terpenoids | P | 385.22 | 0.311±0.009$^{a}$ | 0.254±0.006$^{a}$ | 0±0$^{b}$ |
| 121 | Dihydrofukinolide | Terpenoids | P | 393.22 | 0.17±0.011$^{a}$ | 0.105±0.012a$^{b}$ | 0±0$^{b}$ |
| 122 | Cinncassiol A 19-glucoside | Terpenoids | P | 545.26 | 0.164±0.019$^{a}$ | 0.2±0.027$^{a}$ | 0±0$^{b}$ |
| 123 | Cinncassiol A | Terpenoids | P | 383.2 | 0.319±0.014$^{a}$ | 0.25±0.011$^{a}$ | 0.005±0.001$^{b}$ |
| 124 | Phytolaccasaponin G | Terpenoids | P | 687.38 | 0.038±0.003$^{b}$ | 0.066±0.002$^{a}$ | 0±0$^{c}$ |
| 125 | Ginkgolide J | Terpenoids | N | 423.13 | 0.009±0.002$^{a}$ | 0.007±0.001$^{a}$ | 0±0$^{b}$ |
| 126 | Hyperforin | Terpenoids | P | 537.39 | 0.03±0.001$^{b}$ | 0.033±0.003$^{b}$ | 0.083±0.003$^{a}$ |
| 127 | Curcumin III | Terpenoids | P | 337.14 | 0.07±0.016$^{b}$ | 0.056±0.008$^{b}$ | 0.214±0.032$^{a}$ |

Different lowercase letters indicate significant differences.

Note: "-" means not detected.
